# Beneficial effects of thyroid hormone on adipose inflammation and insulin sensitivity of obese Wistar rats

**DOI:** 10.14814/phy2.13550

**Published:** 2018-02-01

**Authors:** Ana C. Panveloski‐Costa, Caroline Serrano‐Nascimento, Paula Bargi‐Souza, Leonice L. Poyares, Gabriela de S. Viana, Maria T. Nunes

**Affiliations:** ^1^ Department of Physiology and Biophysics Institute of Biomedical Sciences University of São Paulo São Paulo Brazil

**Keywords:** Adipose tissue, cytokines, insulin sensitivity, obesity, triiodothyronine

## Abstract

Thyroid hormones play an important role in glucose metabolism and there is evidence of increased prevalence of thyroid dysfunction in obese and diabetic patients. This study aimed at evaluating the thyroid function and the effects of the triiodothyronine (T3) treatment on glycemia control, insulin sensitivity and subclinical inflammation in cafeteria‐diet‐induced obesity in rats. Obesity was induced in male Wistar rats by offering a cafeteria diet and a subset of the obese rats was treated with T3 (1.5 *μ*g per 100 g of body weight) for a 28‐day period. The pituitary‐thyroid axis was evaluated by molecular and biochemical parameters. Cytokine content was measured in the serum as well as in the mesenteric and epididymal white adipose tissue. Obese rats exhibited impairment of glycemia control, increased content of inflammatory cytokines in mesenteric white adipose tissue, decreased serum thyrotropin (TSH) concentration and increased sodium/iodide symporter (NIS) and TSH receptor (TSHR) protein content in thyroid gland. T3 treatment improved insulin sensitivity, glucose tolerance, and reduced inflammatory cytokine content in mesenteric white adipose tissue. In the thyroid gland NIS, TSHR, and thyroperoxidase (TPO) content were reduced while thyroglobulin (TG) content was increased by T3. The thyrotrophic response to negative feedback exerted by T3 was preserved in obese rats. The present data reinforce the beneficial effects of T3 treatment of obese rats on the improvement of insulin sensitivity and on the negative modulation of inflammatory cytokine expression in adipose tissue. Moreover, we have evidenced that the pituitary‐thyroid axis is affected in obese rats, as illustrated by the impaired TSH secretion.

## Introduction

Although there are reports establishing a relationship between obesity and thyroid disorders, this issue still remains unclear. Reduced thyroid function has been observed in obese individuals (Ruhla et al. 2010; Giandalia et al. 2014). Moreover, increased serum thyrotropin (TSH), elevated triglycerides concentrations, and waist circumference were positively correlated in obese children (Shalitin et al. 2009). More recently, thyroid follicular cell steatosis was associated with the development of primary thyroid failure in obese mice and humans (Lee et al. 2015).

Some studies have also shown high prevalence of thyroid dysfunction in type 1 diabetes mellitus patients (Joffe and Distiller 2014) and rat models (Gonzalez et al. 1980; Rondeel et al. 1992). We have recently detected morphological and molecular alterations, characteristic of primary hypothyroidism, in the thyroid gland of alloxan‐induced diabetic rats (Panveloski‐Costa et al. 2016). Furthermore, we have shown that triiodothyronine (T3) treatment has positive effects on insulin sensitivity in diabetic rats associated with a reduction in inflammatory cytokine expression, decreased hepatic glucose production and increased urinary glucose excretion (Panveloski‐Costa et al. 2016; Teixeira et al. 2016).

Obesity gives rise to a state of chronic, low‐grade inflammation characterized by a dysregulated production and secretion of inflammatory cytokines due to macrophage infiltration into the white adipose tissue (Weisberg et al. 2003; Gregor and Hotamisligil 2011). Indeed, this process is linked to systemic insulin resistance in obese humans and in animal models (Weisberg et al. 2003; Lumeng et al. 2007). Some cytokines such as chemokine (C‐C motif) ligand 2 (CCL2), an important marker of macrophage infiltration, interferon‐gamma (IFN‐*γ*), interleukin‐1 alpha (IL‐1*α*), interleukin‐1 beta (IL‐1*β*), interleukin‐6 (IL‐6), and tumor necrosis factor – alpha (TNF‐*α*) are strongly related to insulin resistance (Weisberg et al. 2003; Gregor and Hotamisligil 2011; O'Rourke et al. 2012).

Considering the correlation between obesity and hypothyroidism, and the benefits of T3 treatment on insulin sensitivity and inflammatory cytokines expression in diabetic rats, the hypothesis of this study was that T3 treatment could induce body weight loss, improvement of insulin sensitivity, and reduction in the obesity‐induced inflammatory state.

## Material and Methods

### Experimental model

Fifty male Wistar rats were maintained on a 12‐h light‐dark cycle (lights on at 7.00 a.m.) at constant temperature (22 ± 1°C) and relative humidity (50–60%) with ad libitum access to water and standard rat chow or cafeteria diet. Food consumption and body weight were recorded weekly. The animals were obtained from the Central Animal Breeding house of the Institute of Biomedical Sciences, University of São Paulo (São Paulo, Brazil). The rats were randomly divided into three groups: Control (C) (nonobese animals), Obese nontreated (O), and Obese T3‐treated (OT_3_).

Obesity was induced in 8‐week‐old rats by a cafeteria diet composed of chow and unhealthy food regularly consumed by humans according to Sampey et al. (2011). The animals whose body weight (BW) was not significantly different from the control group after ingesting the cafeteria diet for 16 weeks were excluded from this study. A subset of obese rats was treated with approximately fivefold the physiological dose of T3 (1.5 *μ*g/100 g BW) (OT_3_) (*n* = 15) or vehicle (Group C and O) (*n* = 15) for a 4‐week period. The dose of T3 used herein was chosen because the physiological dose (0.3 *μ*g/100 g BW) was unable to significantly improve insulin sensitivity and reduce inflammatory cytokine expression (data not shown). In addition, the T3 dose used in this study has been employed in previous studies (Panveloski‐Costa et al. 2016; Teixeira et al. 2016). It is relevant to stress that this dose of T3 treatment did not change the hemodynamic parameters in comparison to the control group.

The rats were euthanized by decapitation after being deeply anesthetized with 80 mg/kg BW of Sodium Pentobarbital (Thiopentax, Cristalia, Itapira, SP, Brazil). Blood was collected from the trunk and the serum obtained was kept at −20°C for biochemical analysis. The target tissues of the study were removed and kept at −80°C for further analysis, as described below. The heart of the animals was collected to determine the heart‐to‐body weight ratio (Kahaly and Dillmann 2005).

This study was approved by the Animal Care and Ethics Committee of the University of São Paulo (106/11, book 2, 2011).

### Insulin (ITT) and Glucose (GTT) Tolerance Test

Rats were fasted overnight for both analyses. ITT was performed using 1 IU of insulin per 1000 g of BW (i.p.), as previously described (Panveloski‐Costa et al. 2016). To evaluate insulin sensitivity, the constant rate for the insulin tolerance test (kITT) was calculated. GTT was performed using 1 g of glucose per 1000 g of BW (45%, i.p.). Glycemia was measured by a glucometer 30, 60, 90, 120, and 150 min after the respective i.p. injections. The area under the curve of glycemia during GTT was calculated in order to evaluate the glucose tolerance status in the animals.

### Triglyceride and cholesterol analysis

Serum triglycerides and cholesterol concentrations were measured by colorimetric reaction according to the manufacturer′s instructions (Labtest SA, Lagoa Santa, MG, Brazil).

### TSH and Thyroid hormone measurement

The total serum T3 and TSH concentrations were quantified by MILIplex assay kit (Millipore Corporation, Billerica, MA, USA) (Elshal and McCoy 2006; Panveloski‐Costa et al. 2016). The total serum thyroxine (T4) concentration was measured by an electrochemiluminescence method, using a commercially available kit (Elecsys Immunoassay kits, Roche Diagnostics, Indianapolis, IN, USA) (Biscolla et al. 2017).

### White adipose tissue (WAT) depots analysis

Epididymal WAT (eWAT) and mesenteric WAT (mWAT) were homogenized using a Polytron PT 2100 (Kinematica AG PT2100) in an appropriate lysis buffer consisting of: 250 mmol/L sucrose, 20 mmol/L Hepes, 5 mmol/L sodium azide, 2 mmol/L EDTA, 10 mmol/L sodium fluoride, 100 mmol/L sodium pyrophosphate, 10 mmol/L sodium orthovanadate, 0.1 mmol/L phenylmethanesulfonyl fluoride and protease inhibitor cocktail (1:100). The homogenate was centrifuged and the supernatant was used to determine total protein concentration by the Bradford method (Bradford 1976) (Bio‐Rad Laboratories, Hercules, CA, USA), as previously described (Panveloski‐Costa et al. 2016). Cytokine content was determined using a MILIplex assay kit (Millipore Corporation, Billerica, MA, USA) (Panveloski‐Costa et al. 2016). The analyzed cytokines were: CCL2, IFN‐*γ*, IL‐1*α*, IL‐1*β*, IL‐6, IL‐10, leptin, and TNF‐*α*.

### Pituitary and Thyroid gland protein expression analysis

Pituitary and thyroid glands were homogenized using a Polytron PT 2100 in RIPA buffer supplemented with a protease inhibitor cocktail (Calil‐Silveira et al. 2016). All buffer components were purchased from Synth (Synth, Diadema, SP, Brazil) and Sigma (Sigma Aldrich, St. Louis, MO, USA). The homogenate was centrifuged and the total protein of the supernatant was determined using the Bradford method (Bradford 1976) (Bio‐Rad Laboratories, Hercules, CA, USA). Immunoblotting of thyroid gland protein extracts was performed using specific antibodies anti‐sodium/iodide symporter (NIS) [kindly provided by Dr. Nancy Carrasco, 1:3000], anti‐TSH receptor (TSHR) [Sigma Aldrich, SAB2102588, 1:1000], anti‐Thyroglobulin (TG) [Abcam, ab80783, 1:5000] and anti‐thyroperoxidase (TPO) [Santa Cruz, sc58432, 1:1000], as previously described (Panveloski‐Costa et al. 2016). Ponceau staining of the nitrocellulose membranes was used to check equal loading of the samples in the gels (Romero‐Calvo et al. 2010; Fortes et al. 2016).

Immunoblotting of the pituitary protein extracts was performed using anti‐rBetaTSH [purchased from Dr. A. Parlow, 1:40000] (Bargi‐Souza et al. 2013). Equal loading was evaluated by incubating the membranes with anti‐GAPDH antibody [Santa Cruz, sc32233, 1:1000].

Secondary peroxidase‐conjugated antibodies (Jackson ImmunoResearch Laboratories, West Grove, PA, USA) and the Enhanced Chemiluminescence kit (Amersham Biosciences, Piscataway, NJ, USA) were used for band detection. Blot densitometry was analyzed using Image J Software (National Institutes of Health, Bethesda, MD, USA). Results are expressed as arbitrary units (AU).

### Reverse transcription and Real‐time quantitative PCR (RT‐qPCR)

Total RNA from the pituitary gland was extracted using TRIzol, according to the manufacturer's instructions (Invitrogen, CA, USA). RNA integrity and RT‐qPCR analysis were performed as previously described (Bargi‐Souza et al. 2013). qPCR was performed using a Platinum SYBR Green quantitative PCR Super Mix UDG and specific primers for *Tshb* (forward: CTCTTCCTGATGGTGAGAAACC; reverse: CCCATGGATGTACAGTAGCAGA) and *Rpl19* (forward: CAATGAAACCAACGAAATCG; reverse: TCAGGCCATCTTTGATCAGCT), which was used as an internal control. Relative changes in gene expression were calculated using the 2^−ΔΔCt^ method (Livak and Schmittgen 2001).

### Statistical analysis

Data are expressed as means ± SEM. The number of animals used in each experiment is indicated in the figures legends. Comparisons between the groups were carried out by one‐way ANOVA, followed by the Student–Newman–Keuls as the post hoc test (GraphPad Prism Software – Version: 5.0 – San Diego, CA, USA).

## Results

### T3 treatment promotes body weight loss of obese rats

**Figure 1:**
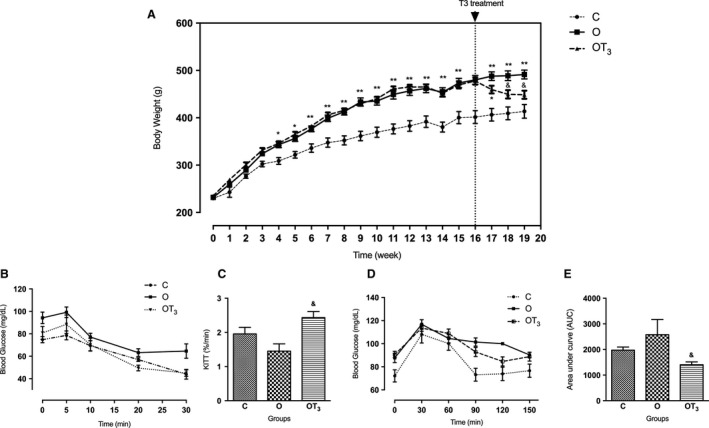
Body weight evolution during experimental protocol (A), insulin tolerance test – ITT (B); rate constant for ITT ‐ kITT (C); glucose tolerance test ‐ GTT (D); area under curve (AUC) for GTT (E) of C (*n* = 10), O (*n* = 12) and OT3 (*n* = 12) rats. Data were analyzed by analysis of variance (one way ANOVA) and Student–Newman–Keuls posttest and expressed as mean ± SEM normalized by total protein content. *P < 0.05 versus C, ** *P* < 0.01 versus C, & *P* < 0.05 versus O.

Figure 1A shows the evolution of the body weight of the animals during the experimental protocol. The cafeteria diet significantly increased the body weight of rats at the fourth week of diet. T3 has significantly reduced the body weight since the first week of treatment.

### T3 treatment improves insulin sensitivity and reduces serum triglycerides and cholesterol concentrations of obese rats

Figure 1 illustrates blood glucose concentration during the ITT (Fig. 1B) and the GTT (Fig. 1D) in Control, Obese and Obese T3‐treated rats. As shown in Figure 1C and E, obese T3‐treated rats presented higher kITT, and a lower area under the curve (AUC) for GTT than the obese rats. Furthermore, T3 treatment reduced the serum levels of triglycerides and cholesterol (Table 1).

**Table 1:** Dry heart weight to body weight ratio (DHW/BW); glycemia; serum triglycerides, cholesterol, TSH, T4, and T3 concentrations in Control (C), Obese (O), and Obese T3‐treated rats (OT_3_)

Parameters	C	O	OT_3_	N per group
DHW/BW	0.65 ± 0.01	0.61 ± 0.01	0.73 ± 0.01&, *	12
Glycemia (mg/dL)	67.0 ± 1.38	95.0 ± 5.15*	84.4 ± 2.21*	10
Triglycerides (mg/dL)	41.0 ± 5.29	71.9 ± 7.25*	51.3 ± 4.07 &	10
Cholesterol (mg/dL)	35.3 ± 3.07	52.6 ± 3.26*	42 ± 3.85 &	10
TSH (pg/mL)	1044 ± 198.7	448 ± 57.9*	137.8 ± 17.4&	12
T4 (pg/mL)	35,687 ± 5,868	46,094 ± 3,285	9,613 ± 2,572&, *	5
T3 (pg/mL)	11,107 ± 761.9	11,804 ± 478.4	11,681 ± 530.1	12

Data were analyzed by analysis of variance (one way ANOVA) and Student–Newman–Keuls posttest and expressed as mean ± SEM.

*P <* *0.05 versus C, ^&^ *P* < 0.05 versus O.

### T3 treatment reduces inflammatory cytokine content in mWAT of obese rats

**Figure 2:**
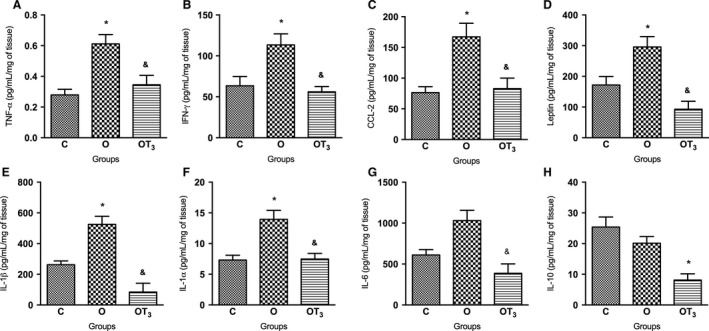
Analysis of TNF‐α (A), IFN‐у (B), CCL‐2 (C), Leptin (D), IL‐1β (E),IL‐1α (F), IL‐6 (G) and IL‐10 (H) concentration in mWAT of C (*n* = 10), O (*n* = 12) and OT3 (*n* = 12) rats. Data were analyzed by analysis of variance (one way ANOVA) and Student–Newman–Keuls posttest and expressed as mean SEM normalized by total protein content. *P < 0.05 versus C, & *P* < 0.05 versus O.

**Figure 3:**
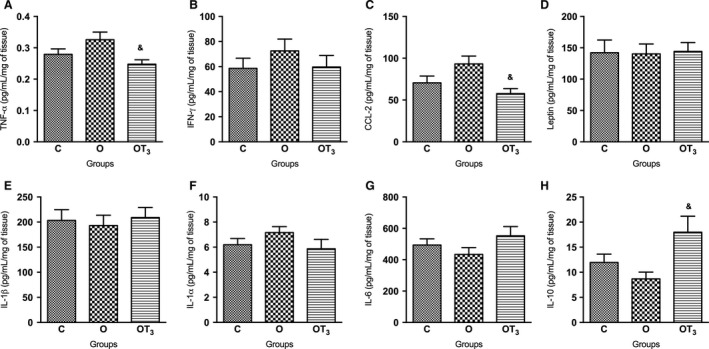
Analysis of TNF‐α (A), IFN‐у (B), CCL‐2 (C), Leptin (D), IL‐1β (E), IL‐1α (F), IL‐6 (G) and IL‐10 (H) concentration in eWAT of C (*n* = 10), O (*n* = 12) and OT3 (*n* = 12) rats. Data were analyzed by analysis of variance (one way ANOVA) and Student–Newman–Keuls posttest and expressed as mean SEM normalized by total protein content. & *P* < 0.05 versus O.

Figures 2 and 3 illustrate the cytokine concentrations in mWAT and eWAT depots, respectively. Obese rats presented an increased expression of all analyzed inflammatory cytokines, except for IL‐6 and IL‐10, in mWAT (Fig. 2). Moreover, the content of these cytokines was reduced in the mWAT of obese T3‐treated rats. There was no significant alteration in the eWAT inflammatory cytokine expression of obese rats in comparison to the control group of rats. However, T3 treatment significantly reduced TNF‐*α* and CCL‐2 content, and increased IL‐10 content in the eWAT of obese rats (Fig. 3).

### Serum leptin concentration increased in obese rats treated or not with T3

**Figure 4:**
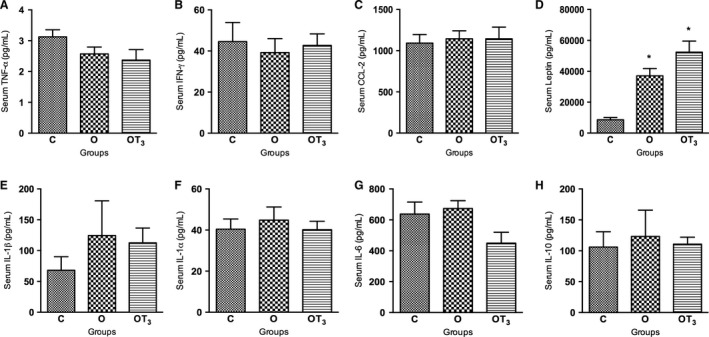
Analysis of serum TNF‐α (A), IFN‐у (B), CCL‐2 (C), Leptin (D), IL‐1β (E),IL‐1α (F), IL‐6 (G) and IL‐10 (H) concentration of C (*n* = 10), O (*n* = 12) and OT3 (*n* = 12) rats. Data were analyzed by analysis of variance (one way ANOVA) and Student–Newman–Keuls posttest and expressed as mean SEM. *P < 0.05 versus C.

As shown in Figure 4, serum TNF‐*α*, INF‐y, CCL‐2, IL‐1*α*, IL‐1*β*, IL‐6, and IL‐10 concentrations were not altered in obese rats in comparison to the control rats. Furthermore, T3 treatment did not alter the serum levels of these inflammatory cytokines in obese T3‐treated rats. On the other hand, serum leptin concentration was significantly increased in nontreated and T3‐treated obese rats (Fig. 4D).

### Obesity reduced TSH secretion, which is compensated by adjustments in thyroid gland function

**Figure 5:**
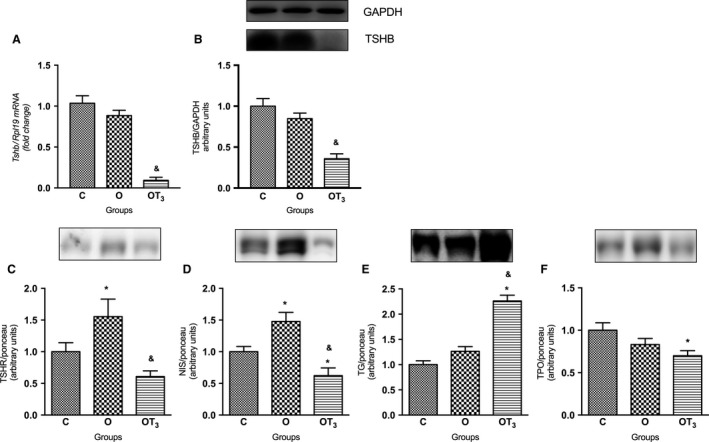
Tshb mRNA expression (A) and TSHB protein content (B) on pituitary; TSHR (C), NIS (D), TG (E) and TPO (F) protein content on thyroid of C (*n* = 10), O (*n* = 10) and OT3 (*n* = 10) rats. Above each graph a typical autoradiogram is shown. Data were analyzed by analysis of variance (one way ANOVA) and Student–Newman–Keuls posttest and expressed as mean ± SEM normalized by Rpl19 mRNA (A), GAPDH content (B), and by Ponceau‐stained membrane – supplementary material (C, D, E, and F). *P < 0.05 versus C, & *P* < 0.05 versus O.

The evaluation of the pituitary‐thyroid axis was performed by biochemical (Table 1) and molecular analysis (Fig. 5). Indeed, obese rats presented lower TSH serum levels in comparison to the control animals (Table 1).

Interestingly, the expression of *Tshb* mRNA (Fig. 5A) and TSHB protein (Fig. 5B) in the pituitary gland were not altered in obese rats in comparison to the control group.

As expected, T3 treatment reduced the TSH serum concentration (Table 1), *Tshb* mRNA (Fig. 5A) and TSHB protein content (Fig. 5B) in the pituitary.

Thyroid protein expression was altered in obese rats, but no significant alterations in T3 and T4 serum levels were observed in these animals (Table 1). In fact, obese rats presented a significant increase in the expression of TSHR (Fig. 5C) and NIS (Fig. 5D) in comparison to control group. Interestingly, T3 treatment reduced the TSHR, NIS and TPO expression in comparison to obese nontreated rats. It is worth noting that T3 treatment increased TG protein expression in the thyroid gland of obese animals (Fig. 5E). Moreover, obese T3‐treated rats presented no significant alterations in T3 serum concentration and reduced serum T4 levels in comparison to the obese nontreated and control groups (Table 1).

## Discussion

This study demonstrates that T3 differentially regulates the expression of inflammatory cytokines in visceral WAT depots of obese rats as well as improves insulin sensitivity and glucose tolerance in these animals.

Cafeteria diet has been demonstrated as a robust model of metabolic syndrome in rats (Sampey et al. 2011). Indeed, the animals subjected to this diet present the common features of obesity, as increased body weight and metabolic alterations, such as increased fasting glycemia, elevated serum triglycerides, and cholesterol concentrations.

The white adipose tissue is a metabolic and endocrine organ, playing an important function on the homeostasis of energy balance (Lopategi et al. 2016). Excessive expansion in obese individuals results in a chronic state of low‐grade inflammation, due to immune cell infiltration and inflammatory cytokine production/secretion by WAT (Weisberg et al. 2003; Gregor and Hotamisligil 2011). These alterations in WAT are highly related to the establishment of insulin resistance and type 2 diabetes mellitus (Hotamisligil 2006).

Accordingly, this study demonstrated an increase in several inflammatory cytokines related to insulin resistance (Weisberg et al. 2003; Gregor and Hotamisligil 2011) in the mWAT, but not in the eWAT, of obese rats, as described for CCL2, an important marker of macrophage infiltration, by Yu et al. (2006). While both adipose tissue depots have been linked to the risk of developing obesity‐related diseases (Bjørndal et al. 2011), the adipose depot‐specific cytokine response to cafeteria‐diet corroborates the assumption that insulin resistance and increased glycemia might be more associated to increased specific fat mass depots than to total body fat mass (Park et al. 2016).

Thyroid hormones exert diverse metabolic actions and, under specific circumstances, their action could promote desirable effects such as increased metabolic rate or lipolysis as well as the reduction in cholesterolemia (Mullur et al. 2014). We have previously demonstrated an important role of T3 treatment reducing the expression of inflammatory cytokines in alloxan‐induced diabetic rats (Panveloski‐Costa et al. 2016). In this context, we hypothesized that T3 treatment could also improve the metabolic disorders induced by obesity condition.

Indeed, the data presented herein indicate that triglyceride and cholesterol levels, insulin sensitivity and glucose tolerance were ameliorated in obese T3‐treated rats. Moreover, body weight gain was decreased in these animals, in agreement with a recent study, which demonstrated that T3 treatment reduces the adiposity and increased the lean mass of obese mice (da Silva Teixeira et al. 2016).

Furthermore, there was a reduction in the TNF‐*α*, IFN‐γ, CCL‐2, IL‐6, IL‐1*β*, IL‐1*α*, IL‐10, and leptin content in the mWAT of obese T3‐treated rats. Conversely, in the eWAT, only the TNF‐*α* and CCL‐2 contents were reduced and the IL‐10 content was increased in response to T3 treatment.

Although T3 has an important role in the control of inflammatory responses (Perrotta et al. 2014), the T3‐induced decrease in adiposity could also contribute to the reduction in cytokines expression, as observed in the mWAT of obese‐treated rats.

It is interesting to note that IL‐10 prevents lipid‐induced insulin resistance (Hong et al. 2009). Moreover, IL‐10 attenuates inflammatory and immune responses through the suppression of inflammatory cytokine production, (Juge‐Aubry et al. 2005). In this study, the T3 treatment induced a reduction in CCL‐2 expression in the eWAT and mWAT of obese rats, which is an important chemokine that regulates the recruitment and maintenance of inflammatory macrophages in adipose tissue (Lumeng et al. 2007). However, there was a different response to T3 treatment in modulating IL‐10 expression in eWAT and mWAT. This difference could be attributed to a possible phenotypic switch in adipose tissue macrophage polarization, once it is known that M1 and M2 macrophages (Perrotta et al. 2014), as well as the distinct adipose tissue depots (Nannipieri et al. 2009) express different thyroid hormone receptors (THRs), of which the THR beta appears to be the major isoform involved in the action of T3 on the macrophages function.

It is really important to mention some immunomodulatory treatments such as salsalate, TNF‐*α* and IL‐1*β* blockers or antagonists of CCL‐2 which target the inhibition of specific cytokines and chemokines, and have been demonstrated to present a beneficial effect on insulin secretion and sensitivity in obese individuals (Donath 2016). In the same way, we have demonstrated herein that T3 also acts as an immunomodulatory agent reducing the content of inflammatory cytokines in the adipose tissue and improving insulin sensitivity of obese rats. These data reinforce that the benefits of T3 administration on glycaemia control of obese rats is associated to the improvement of the inflammatory state, as recently demonstrated in a type‐1 diabetes mellitus model (Panveloski‐Costa et al. 2016).

Serum cytokine concentrations were not altered either by diet‐induced obesity or by T3 treatment, except for leptin levels which were increased both in the obese nontreated and T3‐treated rats. The increment of leptin levels in obese rats was expected (Zhang and Scarpace 2006). Moreover, it has been previously shown that thyroid hormones upregulate leptin expression and secretion in adipocytes (Yoshida et al. 1998). Therefore, the maintenance of elevated serum leptin levels in the mWAT and eWAT of obese T3‐treated rats could be ascribed to the increased secretion of this cytokine by T3 treatment.

In contrast to the higher serum TSH levels observed in type 1 diabetic rats (Panveloski‐Costa et al. 2016), obese rats presented lower levels of this hormone in comparison to nonobese rats. Interestingly, we have not detected alterations in *Tshb* mRNA or TSHB protein contents in the pituitary of obese animals, which strongly suggests an impairment of TSH secretion in obese rats. Considering that TSH secretion is primarily regulated by thyroid hormones, which are within the normal range in obese animals, and by thyrotropin‐releasing hormone (TRH), the alteration in TSH secretion is probably related to an impaired TRH secretion, as observed in other studies of diabetes mellitus models (Rondeel et al. 1992; Van Haasteren et al. 1997).

TSH secretion could be modulated by other factors besides the fluctuation of thyroid hormones in serum and the negative feedback exerted by them (de Moura and de Moura 2004). For instance, it is well described that leptin has a stimulatory effect on the activity of the hypothalamic‐pituitary‐thyroid axis (Ortiga‐Carvalho et al. 2002; Ghamari‐Langroudi et al. 2010). However, in obese conditions, elevated leptin serum levels are commonly related to the reduction in the signaling pathway of this cytokine (Zhang and Scarpace 2006). Therefore, although we have observed increased levels of leptin in the obese rats, the impairment of its signaling pathway could contribute to the reduction in serum TSH levels in these animals.

It is important to emphasize that serum TSH concentrations are higher in the rats of the control group of this study compared to the previous one (Panveloski‐Costa et al. 2016; Teixeira et al. 2016). This result could be related to the different age of the animals. In addition, although increased serum TSH levels has been consistently reported in humans during elderly (Surks and Hollowell 2007; Veltri et al. 2017), the data on this issue in rats are contradictory (Chen and Walfish 1979; Huang et al. 1980; Donda et al. 1987; Gyves et al. 1987; Pekary et al. 1987; Goya et al. 1989; Cizza et al. 1996; Borges et al. 1998) (see further details in the supplemental material).

It is worth noting that reduced TSH levels did not reflect in significant alterations in T3 and T4 levels in the obese rats. This result could be related to a compensatory mechanism in the thyroid gland of obese rats that presented increased expression of TSHR and NIS. It is well described that thyroid hormone production is positively regulated by TSH, which interacts with TSHR, a G‐protein coupled receptor, to exert its stimulatory effects on thyroid (Dremier et al. 2002). Previous studies have shown that TSH negatively regulates the expression of its own receptor in the thyroid gland (Weiss et al. 1997). Therefore, the increased expression of TSHR in obese rats could be explained by the reduced serum TSH levels.

In addition, TSH is the main positive regulator of NIS expression and activity, which is the first and limiting step for thyroid hormones production (Dai et al. 1996; Kogai et al. 1997). Therefore, the increased expression of NIS in the thyroid gland of obese rats could be related to the increased expression of TSHR and, consequently, augmented TSH signaling pathway in the thyrocytes. Taken together, our data suggest that the increased expression of TSHR and NIS in obese rats could maintain normal T4 production, even with reduced TSH serum levels. However, we cannot discard that the persistence of the obese state could lead to a future thyroid failure/impairment and a corresponding increase in TSH levels (Ruhla et al. 2010; Giandalia et al. 2014; Lee et al. 2015).

Lee et al. (2015) reported increased TSHR, NIS, TPO, and TG mRNA content in high‐fat diet‐induced obese mice but, differently of our results, they showed increased TSH levels. It is worth noting that the induction of obesity and the animal models used in their study were distinct from those used in our study. Therefore, different molecular mechanisms could be involved in the regulation of the pituitary‐thyroid axis activity in these different experimental conditions.

Finally, as expected, T3 treatment significantly reduced the concentration of serum TSH and T4 levels in obese rats. Therefore, although obesity reduced TSH secretion, as discussed above, the negative feedback loop exerted by T3 in the pituitary was preserved, since T3 is the main downregulator of TSH synthesis and secretion (Zoeller et al. 2007; Ortiga‐Carvalho et al. 2016; Bargi‐Souza et al. 2017). Furthermore, T3 treatment reduced the thyroid expression of NIS, TSHR and TPO, which are essential for thyroid hormone synthesis. These results could explain the reduced T4 levels that were observed. In addition, T3 treatment increased TG protein content in the thyroid gland of obese rats suggesting an impairment of thyroid hormone secretion, in the same way that was demonstrated for type 1 diabetic rats (Panveloski‐Costa et al. 2016).

Although a supraphysiological dose of T3 was used, serum T3 levels were kept similar to those presented by the obese nontreated rats. This finding was also observed in T3‐treated alloxan‐induced diabetic rats, which presented increased mRNA expression of type‐1 deiodinase in the liver, suggesting an increased conversion of T3 into T2 in this tissue (Panveloski‐Costa et al. 2016).

In brief, this study reinforces the relationship between obesity and thyroid axis disorders as well as the positive effects of T3 treatment improving the glucose and lipid metabolism in this prediabetic condition.

## Conclusion

In conclusion, our results show an important role of T3 treatment on the reduction in the expression of WAT inflammatory cytokines associated to insulin resistance and on the improvement of insulin sensitivity of obese rats. Moreover, this study shows that the obesity condition induces impairment of TSH secretion, an alteration that was compensated by an increased activity of thyroid gland.

## Conflict of Interest

The authors have nothing to disclose.

## Supporting information




**Figure S1.** Ponceau S staining from nitrocellulose membranes generated for TSHR and Tg **(A)** and of NIS and TPO **(B)** detection was used as control loading. Protein samples were obtained from the thyroid gland in Control (C), Obese (O), and T3‐treated obese (OT_3_) rats.
