# Supplementary material for: Beneficial effects of thyroid hormone on adipose inflammation and insulin sensitivity of obese Wistar rats
Source: Physiol Rep. 2018 Feb 1;6(3):e13550. doi: 10.14814/phy2.13550 (PMC5817825; doi:10.14814/phy2.13550)
Supplement: Supplementary file 1 — Figure S1. Ponceau S staining from nitrocellulose membranes generated for TSHR and Tg (A) and of NIS and TPO (B) detection was used as control loading. Protein samples were obtained from the thyroid gland in Control (C), Obese (O), and T3‐treated obese (OT3) rats. [file PHY2-6-e13550-s001.docx]

**SUPPLEMENTAL MATERIAL OF FIGURE 5**

**
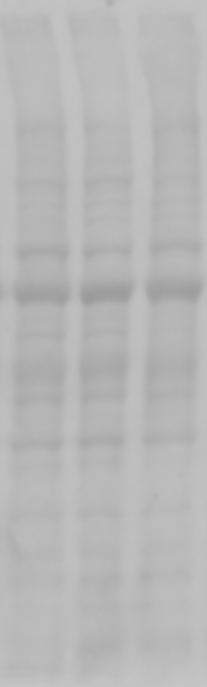

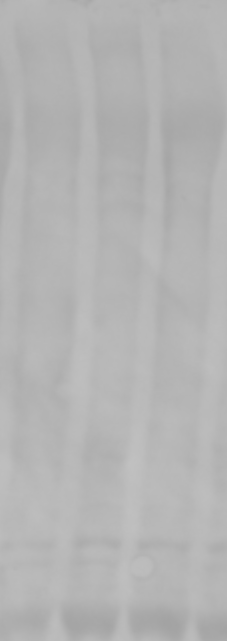
A B**

**C O OT_3_**

**C O OT_3_**

**Figure S.** Ponceau S staining from nitrocellulose membranes generated for TSHR and Tg **(A)** and of NIS and TPO **(B)** detection was used as control loading. Protein samples were obtained from the thyroid gland in Control (C), Obese (O) and T3-treated obese (OT_3_) rats.

**SUPPLEMENTAL MATERIAL OF DISCUSSION**

Serum TSH concentrations are higher in the rats of the control group of the present study compared to the previous one (Panveloski-Costa et al. 2016, Teixeira et al. 2016), and this could be due to the different age of the animals. In fact an increase of serum TSH levels with aging has been consistently reported in humans (Surks et al. 2007, Veltri et al. 2017). However the data on this issue in rats are contradictory, since some studies show no difference in serum TSH levels between young and old rats (Huang et al. 1980, Donda et al. 1987), and others reported decreased (Pekary et al. 1987, Borges et al. 1998) or increased serum TSH levels in old rats (Chen and Walfish 1979, Gyves et al. 1987, Goya et al. 1989). Moreover, it has been reported that with aging large molecular forms of TSH are present in the serum, and it is possible that they could be measured in the assay leading to a false increase in TSH (Gyves et al. 1987). In addition, these larger TSH molecules are supposed to be cleared more slowly from the circulation. It is interesting to comment that stress leads to a decrease in serum TSH levels and this effect is clearly detected in young, but not in old rats (Cizza et al. 1996).

References:

1. Borges PP, Curty FH, Pazos-Moura CC, Moura EG. 1998. Effect of testosterone propionate treatment on thyrotropin secretion of young and old rats in vitro. *Life Sci*, **62**: 2035-2043.
2. Chen HJ, Walfish PG. 1979. Effects of age and testicular function on the pituitary-thyroid system in male rats. *J Endocrinol*, **82**: 53-59.
3. Cizza G, Brady LS, Esclapes ME, Blackman MR, Gold PW, Chrousos GP. 1996. Age and gender influence basal and stress-modulated hypothalamic-pituitary-thyroidal function in Fischer 344/N rats. *Neuroendocrinol*, **64**: 440-448.
4. Donda A, Reymond MJ, Zürich MG, Lemarchand-Béraud T. 1987. Influence of sex and age on T3 receptors and T3 concentration in the pituitary gland of the rat: consequences on TSH secretion. *Mol Cell Endocrinol*, **54**: 29-34.
5. Goya RG, Quigley KL, Takahashi S, Reichhart R, Meites J. 1989. Differential effect of homeostatic thymus hormone on plasma thyrotropin and growth hormone in young and old rats. *Mech Ageing Dev*, **49**: 119-128.
6. Gyves PW, Gesundheit N, Taylor T, Butler JB, Weintraub BD. 1987. Changes in thyrotropin (TSH) carbohydrate structure and response to TSH-releasing hormone during postnatal ontogeny: analysis by concanavalin-A chromatography. *Endocrinol*, **121**:133-140.
7. Panveloski-Costa AC, Silva Teixeira S, Ribeiro IM, Serrano-Nascimento C, das Neves RX, Favaro RR, et al. 2016. Thyroid hormone reduces inflammatory cytokines improving glycaemia control in alloxan-induced diabetic wistar rats. *Acta Physiol (Oxf)*, **217**:130-140.
8. Pekary AE, Mirell CJ, Turner LF Jr, Walfish PG, Hershman JM. 1987. Hypothalamic secretion of thyrotropin releasing hormone declines in aging rats. *J Gerontol*, **42**: 447-450.
9. Surks MI, Hollowell JG. 2007. Age-specific distribution of serum thyrotropin and antithyroid antibodies in the US population: implications for the prevalence of subclinical hypothyroidism. *J Clin Endocrinol Metab*, **92**: 4575-4582.
10. Teixeira SD, Panveloski-Costa AC, Carvalho A, Monteiro Schiavon FP, Ruiz Marque AC, Campello RS, et al. 2016. Thyroid hormone treatment decreases hepatic glucose production and renal reabsorption of glucose in alloxan-induced diabetic Wistar rats. *Physiol Rep*, **4**: p.e12961.
11. Veltri F, Rocha FO, Willems D, Praet JP, Grabczan L, Kleynen P, et al. 2017. Prevalence of thyroid dysfunction and autoimmunity in the older population and implications of age-specific reference ranges. *Clin Chim Acta*, **465**: 34-39.
